# Do Visionary-Feedback Seeking CEOs Enhance Firm Sustainability Through Eco-Innovation? A Moderated Mediation Model

**DOI:** 10.3389/fpsyg.2021.750885

**Published:** 2022-01-31

**Authors:** Cheng Yuanyuan, Farzan Yahya, Muhammad Waqas, Li Hongbo

**Affiliations:** ^1^School of Management, Jiangsu University, Zhenjiang, China; ^2^School of Finance and Business, Zhenjiang College, Zhenjiang, China; ^3^Institute of Southern Punjab, Multan, Pakistan; ^4^Department of Business Administration, Institute of Southern Punjab, Multan, Pakistan

**Keywords:** CEO, vision articulation, feedback-seeking, eco-innovation, sustainability, TMT, boundary-spanning

## Abstract

Based on upper echelons, paradox, and social capital theory, this study extends the association of CEO vision articulation and feedback-seeking behavior with firm sustainability by identifying the mediating role of eco-innovation and top management team (TMT) boundary-spanning behavior as a moderator. By analyzing the data of mid-sized to large Chinese firms using hierarchical regression and bootstrapping-based moderated path analysis, we found that product and process eco-innovation mediates the link between CEO vision articulation and firm sustainability while CEO feedback-seeking behavior enhances firm’s sustainability through product eco-innovation only. Finally, conditional indirect effects show the vital role of TMT boundary-spanning behavior in facilitating CEOs to improve the firm’s long-term sustainability through eco-innovation.

## Introduction

Around the globe, firms are continuously making efforts to mitigate the environmental impact of their economic activities to deal with public awareness for green products and climate-friendly technology (Kopnina and Blewitt, 2014). However, balancing environmental concerns with profit-driven development is essential for long-term firm survival (Du et al., 2012). Thus, environmental innovation is emerged as a new mechanism to achieve firm sustainability (Liao, 2016; Polzin et al., 2016). Eco-innovation; defined as the development of products and processes that contribute to sustainable development (Rennings, 2000), differs from the conventional innovation process as it improves green performance through environmental externalities and positive R&D spillover (Bossle et al., 2016; Hojnik and Ruzzier, 2016). It also acts as a powerful mechanism to remove information asymmetries among market players and facilitates enterprises to comply with stringent environmental regulations, which eventually leads to the firm’s sustainability (Przychodzen and Przychodzen, 2015; Kuo and Smith, 2018; Santos et al., 2019). To date, a wide range of regulatory drivers and demand-supply side drivers of eco-innovation are investigated, which pushes firms toward internalizing external costs (Jové-Llopis and Segarra-Blasco, 2018; Kuo and Smith, 2018). Another strand of studies has investigated the effect of stakeholders’ pressure and environmental policy on environmental performance (Doran and Ryan, 2016; Yu et al., 2017). Nonetheless, researchers have devoted excessive attention to the technological, market, and environmental aspects while ignoring the individual and organizational factors. As a result, the domain of eco-innovation is still in its infancy (Liao and Long, 2018).

Based on the review of prior literature, we identified certain gaps to complement current studies in eco-innovation. First, there is a general dearth of evidence related to the association of eco-innovation and firm sustainability as most of the studies are inclined toward firm performance with mixed views (Doran and Ryan, 2016; Tang et al., 2018; Andries and Stephan, 2019). Even though eco-innovation is important to achieve sustainability (Kuo and Smith, 2018; Mazzanti, 2018), empirical research has ignored this perspective. Second, based on the upper echelon notion, researchers argue that different CEOs’ capabilities can help organizations achieve competitive advantage and sustainability (Huang et al., 2019; Shahab et al., 2020). Still, there are undetermined CEO characteristics and underexplored mediating factors through which CEOs improve their firm’s sustainability.

Although a few studies addressed the importance of CEO vision articulation and feedback-seeking on firm sustainability (Ashford et al., 2016, 2018), our research extends this literature by demonstrating that CEOs who evolve and articulate a vibrant and tempting vision, fosters perception of value congruence between employees and organizational goals (Dvir et al., 2004; Ashford et al., 2018). Followers perceive such CEOs as vibrant, capable, high-spirited, and confident (Grant, 2012; Ehlen et al., 2018). Nevertheless, Ashford et al. (2018) revealed that the humble CEOs who seek feedback could also improve firm performance even if they are not projected as visionary leaders.

Humble CEOs more effectively share information and collaborate with their top management team (TMT) by adopting an ambidextrous strategic orientation (Ou et al., 2018). Nonetheless, there is little theoretical understanding of the vision articulation and feedback-seeking behavior of the CEOs in explaining the process and product eco-innovation. Although it is articulated that CEO vision articulation and feedback-seeking behavior independently enhance eco-innovation for corporate sustainability, the interaction of these two characteristics may provide captivating estimates. Using a paradox perspective, it is posited that contradictory traits can co-exist in an individual. The interaction of CEO vision articulation (less humble) and feedback-seeking (more humble) behavior may create a social charisma that stimulates innovative culture in an organization (Zhang et al., 2017).

Besides the paradox perspective, the co-existence of opposite elements can be explained by “cultural additivity.” The term was initially coined by Klug (1973) and further explored by Vuong et al. (2018). It is a mechanism whereby individuals acquire values that might or might not logically contradict their core values. Altruism and humility is a virtue and integral part of Chinese culture backed by the Confucian perspective (Li, 2016). Thus, less humble CEOs have to adopt a humble attitude to survive in the executive-level positions in China. Regardless of any other dominant trait, the primordial needs of humility for Chinese leaders spark employee proactivity and empowerment (Chen et al., 2018), triggering sustainable innovation (Throop and Mayberry, 2017).

Third, we argue that TMT boundary-spanning behavior strengthens the mediating effect of eco-innovation between underlying CEO characteristics and firm sustainability. The seminal work on team boundary-spanning behavior has considered three dimensions, i.e., ambassador activities, task coordinator activities, and scout activities (Ancona and Caldwell, 1992). Through these activities, TMT communicates vigilantly with the higher department to attain support, collect technical information from external resources, explore market opportunities, monitor competitors’ activities, and resolve operational issues by working together with their external partners. Thus, there is a positive effect of TMT boundary-spanning behavior on the innovative activities of the firm (Yan et al., 2020). Nonetheless, almost no empirical study has reported the moderating role of TMT boundary-spanning behavior to advance the theory in this area.

**FIGURE 1:**
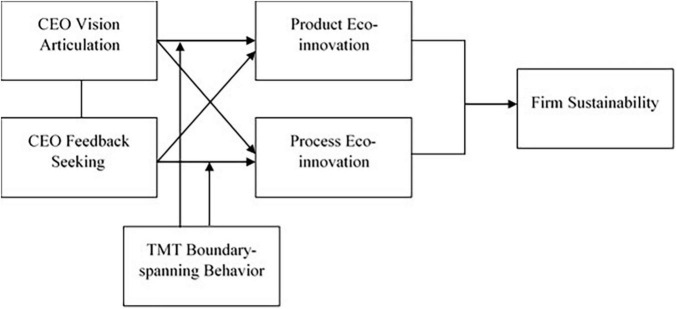
The proposed moderated mediation model.

In summary, our study contributes to the upper echelon, sustainability, and eco-innovation literature by addressing and statistically investigating a moderated mediation model in which eco-innovation intervenes in the effect of the CEO leadership qualities (vision articulation and feedback-seeking) and TMT boundary-spanning behavior on firm sustainability. The research model of the study is shown in Figure 1.

## Theory and Hypotheses

Eco-innovation may include several activities such as clean energy utilization, green patents, environmentally responsive management systems, green technology, recycling, and so on (Park et al., 2017). Eco-innovation is essential for developing synergies between competitiveness and sustainability toward a green economy (Mazzanti, 2018). While discussing the drivers of eco-innovation, researchers argue that green innovation is a matter of managerial decision-making rather than any definite business policy (Tang et al., 2018). However, these activities can be divided into green products and process innovation. In green product innovation, firms produce products using recycling or green material while clean energy resources are utilized in the manufacturing process to reduce environmental pollution (Ma et al., 2017; Xie et al., 2019). It is important to differentiate these types of eco-innovation, as they are usually driven by the different supply sides, demand sides, and regulatory factors. The eco-process innovation (productive efficiency) is reinforced by supply-side drivers such as cost-saving or technology push. On the other hand, eco-product innovation (product quality) is determined by demand-side factors including the market share and market demand for green products (Triguero et al., 2013). Owing to the distinct characteristics of radical and incremental eco-innovation at the firm-level (Carrillo-Hermosilla et al., 2010), we segregated the eco-innovation into product and process innovation.

Taking a step forward, based on the upper echelons theory (UET), organizational performance and behaviors are not merely dependent upon firm value or external environment but also the result of top management’s traits and cognitive capabilities (Hambrick and Mason, 1984). Over the past few years, UET has served as a catalyst for investigating how top management or executives’ traits and characteristics shape their actions, choices, and perceptions that eventually influence various forms of organizational outcomes (Ashford et al., 2018; Neely et al., 2020). Nonetheless, there is relatively scarce literature on how CEOs’ leadership or behavioral characteristics influence environmental product and process innovation (Liao and Long, 2018). CEOs assess an organization’s capability to achieve a competitive advantage if they are articulating a vision for the firm. Top management teams consider vision articulation behavior as a dynamic role of charismatic leadership which leads to improved firm and environmental performance (Wowak et al., 2016; Ashford et al., 2018).

Along with Stam et al. (2014) and Krug et al. (2020), we define vision articulation as “the ability to motivate followers to contribute to the realization of the vision.” Vision articulation is one of the essential components of charismatic or transformational leadership and the term is interchangeably used as “visionary leadership” in previous studies (Krug et al., 2020). The vision articulation behavior in leaders is generally associated with stronger stakeholder values and diverts organizational attention toward sustainable goals (Metcalf and Benn, 2013). CEO’s ability to communicate an idealized vision is vital to mitigate the environmental effects of corporate processes and their products by allocating the firm’s budget for eco-friendly projects (Zhou et al., 2018). Considering the traits of charismatic or transformational leadership, researchers argue that vision articulation of a leader generates socialized charisma, which leads to superior, innovative activities (Arena et al., 2018; Sattayaraksa and Boon-itt, 2018). The ability of a CEO to articulate vision reinforces organizational identity and shared vision (Gioia et al., 2010) which strengthens the sense of psychological ownership of the firm’s green products (Chang, 2020). Visionary leaders develop an adhocracy culture in the organization through which they encourage an entrepreneurial environment, new idea generation, and encourage risk-taking that eventually boost eco-innovation (del Rosario and René, 2017). Thus, we argue that CEOs with stronger vision articulation capability enhance the type of innovation which can engender stakeholder values and mitigate environmental degradation at the same time, leading firms to long-term sustainability.

H_1_: *Product eco-innovation mediates the relationship between CEO vision articulation and firm sustainability.*

H_2_: *Process eco-innovation mediates the relationship between CEO vision articulation and firm sustainability.*

Recently, considerable research is devoted to the leader’s humility and its influence on firm innovation, firm performance, and team effectiveness (Zhang et al., 2017; Ou et al., 2018; Rego and Simpson, 2018). Nonetheless, there is an inadequate understanding of specific humble behaviors and characteristics that enhance the firm’s sustainability. Among the various traits of humble leadership, Ashford et al. (2018) isolated the unique effects of feedback-seeking behavior of CEO from humility and argued that the CEOs who seek the feedback of their behavior and performance boost TMT potency, which eventually enhances the firm’s sustainability.

Feedback is considered as a dynamic process that consists of a feedback receiver’s perception, feedback information quality, and feedback source credibility (Dahling et al., 2017). All these activities support the feedback-seeking results through which leaders improve employees’ and team creativity to develop innovative behavior in employees (Su et al., 2019). Although prior studies have not specifically discussed how the feedback-seeking behavior of leaders may influence environmental concerns of the organization, Jones Christensen et al. (2014) asserted that humble or servant leaders might have concerns for others due to which they enhance the social and environmental sustainability of the firm. Additionally, feedback-seeking behavior acts as an agile learning strategy for sustainable careers (Anseel, 2017). Thus, we propose that CEOs who seek feedback on their behavior may improve firm sustainability by promoting green products and technologies to account for the environmental concerns of society. Accordingly, the following hypotheses are developed:

H_3_: *Product eco-innovation mediates the relationship between CEO feedback-seeking behavior and firm sustainability*.

H_4_: *Process eco-innovation mediates the relationship between CEO feedback-seeking behavior and firm sustainability*.

The framework of upper echelons theory incorporates the role of top management teams who are responsible for strategic change, adaption, and organizational growth. The efficient top management team favors creativity, an idea generation environment, effective communication, and a cooperative climate which improves product and process innovation performance (Ruiz-Jiménez et al., 2016; Jiao et al., 2019). Correspondently, TMT boundary-spanning behavior can be considered as a flexible and cost-effective way to attain external resources (Yan et al., 2020). Consistent with social capital theory, researchers argue that TMTs promote organizational learning through their external social capitals, social networks, and a high level of network centrality through which firm innovation capability can be improved (Li et al., 2014; Jiao et al., 2019). Thus, Yan et al. (2020) revealed that TMT boundary-spanning is positively associated with business model innovation in Chinese SMEs.

TMT boundary-spanning functions through the ambassador, task coordinator, and scout activities. Top management teams coordinate with the CEO, board, and other higher departments to gain trust and appreciation through ambassador activities. TMTs solve institutional, operational, and strategic issues by creating an alliance with their external human resources through task coordinator activities. Furthermore, top management teams attain technical information, reconnoiter market prospects, and oversee similar projects of their competitors through scout activities. By gaining resource support from internal and external stakeholders, TMT boundary-spanning behavior may support CEO’s vision to improve product and process eco-innovation to meet sustainability needs of the firm as TMT members tend to have a more sensitive market perception, vigorous sense of vision, and broader vision (Yan et al., 2020).

TMT members build effective lateral communication networks with external stakeholders by holding outside directorships (Jiao et al., 2019) that promise informational benefits (Ferguson et al., 2019). Thereby informing CEOs to broader their CEO’s vision to galvanize green agenda into action and promote green innovation in the organization. Additionally, CEOs who display more feedback-seeking behavior receive more useful, supportive, and information from coworkers (Zhou, 2003; Anseel et al., 2015). TMTs with more external linkages may more likely identify themselves with their organization under a feedback-seeking environment (Ashford et al., 2016; De Stobbeleir et al., 2020) and thrive to stimulate green organizational performance (Cao and Chen, 2019). Thus, in both cases (CEO vision articulation and feedback-seeking), TMT boundary-spanning behavior facilitates CEOs to pursue stakeholders’ interests through environmental innovation. These homogenized TMT-CEO efforts foster firm’s long-term sustainability. Accordingly, we hypothesize the following:

H_5_: *TMT boundary spanning behavior moderates the positive indirect relationship between CEO feedback-seeking behavior and firm sustainability through product eco-innovation such that the link is stronger when the TMT boundary spanning behavior is high*.

H_6_: *TMT boundary spanning behavior moderates the positive indirect relationship between CEO feedback-seeking behavior and firm sustainability through process eco-innovation such that the link is stronger when the TMT boundary spanning behavior is high.*

H_7_: *TMT boundary spanning behavior moderates the indirect positive relationship between CEO vision articulation and firm sustainability through product eco-innovation such that the link is stronger when the TMT boundary spanning behavior is high.*

H_8_: *TMT boundary spanning behavior moderates the indirect positive relationship between CEO vision articulation and firm sustainability through product process eco-innovation such that the link is stronger when the TMT boundary spanning behavior is high*.

## Research Methodology

### Sample and Data Collection Procedure

Survey data were collected from TMT members (identified by their CEO) of 91 mid-sized to large enterprises comprised of 8 different industries located in China. Primarily, we contacted 284 TMT members for their participation in the survey (94 from large enterprises and 190 medium enterprises). Some of the TMT members did not respond, while others denied sharing information due to their organizational policy. After discarding missing data, a final sample of 234 TMT members was considered. Major industries were related to fertilizer, transportation, automobile, energy supply, sugar, cement, retail, and paperboard. The average age of sample firms was 28.7 years (*SD* = 22.80) with moderate tenure of CEO (*M* = 6.4 years, *SD* = 5.1).

Before the distribution of surveys, we conducted a pilot study by randomly choosing TMT members from different firms to ensure that the content of the questionnaire is comprehendible. Common method variance bias is mitigated through the method proposed by Podsakoff et al. (2003). Firstly, we assured the participants of anonymity and confidentiality to mitigate evaluation apprehension at the reporting stage. Secondly, Harman’s one-factor test was used. The results show that common method variance bias is not a serious issue in the study as the strongest factor accounted for 17.22 percent of the total variance, whereas other factors explained 82.78 with an eigenvalue greater than 1.00.

### Measures

#### Firm Sustainability

The five-item scale developed by Harmon et al. (2010) was used to measure the effectiveness of the firm’s sustainable practices. This comprehensive scale is appropriate for measuring just and fair sustainability practices and principles of an organization in both the short and long run. Items were scored on a five-point scale ranging from 1 (strongly disagree) to 5 (strongly agree). Karkoulian et al. (2016) estimated the appreciable reliability of the scale (α = 0.918) in their study. Likewise, we also evaluated the good reliability of the scale (α = 0.881).

#### CEO Vision Articulation

We used the scale developed by House et al. (2013) to measure CEO vision articulation. The two-item scale reflects the perceptions of TMT members related to their CEO’s vision for prospective growth. The items include “the CEO has a vision and imagination of the future” and “the CEO has a clear sense of where he/she wants this organization to be in 5 years,” Consistent with the estimations of Ashford et al. (2018), we also found a high correlation between the items of this scale (*r* = 0.78).^1^

#### CEO Feedback Seeking Behavior

CEO feedback-seeking behavior was measured using the scale developed by Ashford and Tsui (1991). This three-item scale assesses the TMT perceptions regarding the persistence of their CEO’s feedback-seeking behavior over the past 6 months. The items include “the CEO directly asks for an informal appraisal,” “the CEO directly asks for information concerning his or her performance,” and “Directly ask you, how am I doing?” The Cronbach’s alpha of the scale was 0.842.

#### Eco-Innovation

The measurement of process and product eco-innovation was adapted from the studies of Utterback and Abernathy (1975); Chen et al. (2006), and Cheng and Shiu (2012). Liao and Long (2018) also utilized this six-item scale in their study, from which three items are related to product eco-innovation while the rest of the items are associated with process eco-innovation. Cronbach’s alpha of the scale was 0.81 for product eco-innovation and 0.75 for process eco-innovation.

#### Top Management Team Boundary-Spanning Behavior

We measured TMT boundary-spanning behavior through the modified scale of Yan et al. (2020), which was originally developed by Ancona and Caldwell (1992). Based on the ambassador, task coordinator, and scout activities of TMT boundary-spanning behavior, the nine-item scale is utilized (three items for each activity). The good reliability of the overall scale is assessed (α = 0.914). All instruments were translated into Mandarin Chinese using a back-translation procedure before survey distribution.

#### Control Variables

The incorporation of control variables in the hierarchical regression model does not assure the elimination of biases that emerge between predictor and explanatory variables regardless of their extensive use in business and organizational research (Carlson and Wu, 2012). Thus, the role of the control variable should be explicit instead of implicit (Spector and Brannick, 2011). We have utilized CEO age, gender, tenure, TMT size, and firm age to control the effect of independent variables on dependent variables in the moderation mediation analysis. In Asian culture, more experienced and older CEOs are highly respected, but they may show much flexibility toward innovation and change (Musteen et al., 2006; Makri and Scandura, 2010). We transformed CEO age and tenure using a natural logarithm to avoid any non-normality issues (Prasad and Junni, 2016).

Similarly, CEO gender is also one of the important factors that influence a firm’s major decisions. Studies revealed that female CEOs are risk-averse and less likely to enhance the firm’s innovative activities (Faccio et al., 2016; Skała and Weill, 2018). To measure the effectiveness of the CEO gender, we coded the value “1” for males and “0” for females. Furthermore, a wide range of studies has considered the influence of TMT size on organizational innovation (Chen et al., 2016; Prasad and Junni, 2016). TMT size is also measured using the natural logarithm of the number of top management teams identified by their CEO. Finally, prior studies argued that younger firms tend to have more risk-taking behavior and invest extensively in R&D activities to improve firm innovation (Coad et al., 2016). A natural log of the number of years since the incorporation of the parent company is used to measure firm age.

### Measure Validation, Aggregation Issues, and Estimation Method

We used abbreviated scales because CEOs or top managers may not respond adequately to lengthy or repetitive questions (Stoker et al., 2012). Although this approach is effective to enhance the response rate, it may raise concerns related to scales’ validity. Thus, ensuring the reliability and validity of the instruments is important for the current study despite their acceptable validation in the prior studies. We proceed with confirmatory factor analysis to assess the measurement validity. Prior studies show that the multiple-factor model generates better indices compared to the one-factor model. Thus, the indices for the six-factor model included the following: χ^2^/df = 2.78, Confirmatory Fit Index (CFI) = 0.92, Goodness-of-fit Index (GFI) = 0.95, Tucker–Lewis Index (TLI) = 0.90, Root Mean Square Error of Approximation (RMSEA) = 0.05. Furthermore, the validity indices of moderated mediation model also show good fit: χ^2^/df = 2.91, Confirmatory Fit Index (CFI) = 0.94, Goodness-of-fit Index (GFI) = 0.93, Tucker–Lewis Index (TLI) = 0.91, Root Mean Square Error of Approximation (RMSEA) = 0.06.

After validating the fitness of our measurements, we proceed with the aggregation of individual responses to the organizational level due to the hierarchical structure of our data. The inter-member reliability coefficients [ICC1, ICC2, mean r_*wg(j*)_] are evaluated for each construct while the *F*-tests are utilized from a series of one-way ANOVAs to evaluate if the average rating of TMT members is significantly different across firms. After excluding two firms with only one TMT member, the results of analyses show that there is considerable consistency among survey responses from members of the same firm as the *F*-test indicates significant results and the values ICC1, ICC2, and r_*wg(j)*_ exceeds the suggested threshold level (Krasikova and LeBreton, 2019).

To test the hypotheses of our moderated mediation model, we analyze multiple regression models hierarchically as suggested by prior studies (Baron and Kenny, 1986; Cohen et al., 2013). We also investigate the significance of the conditional indirect effects using bootstrapping-based moderated path analysis (Edwards and Lambert, 2007).

## Results

Table 1 shows mean, standard deviation, correlations and Cronbach’s alpha of all variables considered in the study. CEO feedback-seeking behavior (*r* = 0.18, *p* = 0.008), process eco-innovation (*r* = 0.34, *p* = 0.023), and TMT boundary-spanning behavior (*r* = 0.27, *p* = 0.000) were positively correlated with firm sustainability. CEO vision articulation was significantly associated with product eco-innovation (*r* = 0.15, *p* = 0.005) while CEO feedback-seeking behavior was positively associated with both product (*r* = 0.11, *p* = 0.007) and process eco-innovation (*r* = 0.29, *p* = 0.042).

**TABLE 1:** Descriptive statistics and correlations.

Variables	Mean	*SD*	1	2	3	4	5	6
Firm sustainability	2.83	1.03	0.88					
CEO vision articulation	3.22	0.83	0.14	0.78				
CEO feedback seeking	2.73	0.94	0.18**	–0.09	0.84			
Product eco-innovation	2.88	1.28	0.22	0.15**	0.11**	0.81		
Process eco-innovation	3.17	1.24	0.34*	0.07	0.29*	0.52*	0.75	
TMT boundary-spanning	3.56	1.17	0.27***	0.16**	0.35	0.25***	0.37*	0.91

*Cronbach’s α reliability coefficients appear on the diagonal.*

*p < 0.05; **p < 0.01; ***p < 0.001, two-tailed test.

To test the H_1_ and H_2_, after controlling the effect of CEO age, gender, tenure, TMT size, and firm age, we entered CEO vision articulation (independent variable) in Model 2 and eco-innovation (mediating variable) in Model 3 and 4 (see Table 2). The direct effect of CEO vision articulation was positively significant with firm sustainability (β = 0.16, *p* < 0.05) in Model 2 but insignificant when product eco-innovation (β = 0.18, *p* < 0.05) and process eco-innovation (β = 0.16, *p* < 0.05) were introduced in the Model 3 and 4 which provided support for our first and second hypothesis as the effect of CEO vision articulation was also positively significant on product and process eco-innovation (see Table 3).

**TABLE 2:** Hierarchical regressions on firm sustainability.

	1	2	3	4	5	6	7	8	9	10
CEO age	0.12	0.10	0.08	0.07	0.08	0.07	0.10	0.09	0.10	0.09
CEO gender	0.05*	0.04	0.02	0.02	0.03	0.02	0.03	0.03	0.02	0.03
CEO tenure	0.19	0.11	0.13	0.11	0.11	0.12	0.11	0.12	0.13	0.11
TMT size	0.38**	0.31*	0.33*	0.30*	0.29*	0.30*	0.31**	0.29**	0.30**	0.27**
Firm age	0.03**	0.02*	0.01	0.01*	0.02	0.01	0.01	0.02	0.01	0.01
CEO vision articulation (CVA)		0.16*	0.18	0.16				0.16	0.17	0.15
CEO feedback seeking (CFS)					0.23**	0.13**	0.14*	0.12**	0.07**	0.04**
Product eco-innovation (PCEI)			0.08*			0.10*		0.07*	0.05**	0.06*
Process eco-innovation (PDEI)				0.10**			0.12	0.09*	0.07**	0.09**
TMT boundary-spanning (TBS)								0.19	0.17*	0.15*
CVA * CFS									0.07*	0.08**
CVA * TBS										0.01
CFS * TBS										0.03*
Adjusted *R*^2^	0.12	0.14	0.18	0.17	0.16	0.21	0.23	0.27	0.29	0.31
Δ*R*^2^		0.02	0.04	0.03	0.04	0.05	0.07	0.15	0.02	0.02
F	30.22**	14.63***	18.85**	19.03**	17.95**	18.72**	15.90**	13.92**	10.03*	9.82**

*p < 0.05; **p < 0.01; ***p < 0.001.

**TABLE 3:** Hierarchical regressions on product and process eco-innovation.

	Product eco-innovation			Process eco-innovation		
	Model 11	Model 12	Model 13	Model 14	Model 15	Model 16
CEO age	−0.07*	–0.08	–0.06	–0.11	–0.10	–0.08
CEO gender	0.02*	0.01**	0.02*	0.05*	0.03*	0.02
CEO tenure	0.09	0.04	0.03	0.12*	0.09*	0.07**
TMT size	−0.23**	−0.21*	−0.20*	0.30	0.27	0.26
Firm age	0.06	0.04	0.05	0.04*	0.02	0.01
CEO vision articulation (CVA)		0.22**	0.17*		0.28	0.25*
CEO feedback seeking (CFS)		0.15**	0.16**		0.14**	0.15*
TMT boundary-spanning (TBS)		0.09*	0.05**		0.12**	0.10***
CVA * CFS			0.04			0.05**
CVA * TBS			0.01			0.06*
CFS * TBS			0.06**			0.02**
Adjusted *R*^2^	0.09	0.17	0.20	0.13	0.18	0.21
Δ*R*^2^		0.08	0.03		0.05	0.03
F	14.28**	11.17***	8.59**	18.81**	9.63**	7.06**

*p < 0.05; **p < 0.01; ***p < 0.001.

On the other hand, CEO feedback-seeking behavior (β = 0.13, *p* < 0.01) and product eco-innovation (β = 0.10, *p* < 0.05) were positively associated with firm sustainability in Model 6 and the effect of CEO feedback-seeking behavior is also positively significant on product eco-innovation (β = 0.16, *p* < 0.01) in Table 3. It shows that product eco-innovation partially mediates the association between CEO feedback-seeking behavior and firm sustainability as the effect size was reduced compared to Model 5. Nonetheless, we did not find any significant association of process eco-innovation with firm sustainability (β = 0.12, *p* > 0.05) when it was incorporated with CEO feedback-seeking behavior in Model 6. Thus, H_4_ was not supported.

To test H_5_ and H_6_, conditional indirect effects using bootstrapping-based moderated path analysis are used. Table 4 shows that the indirect effect of CEO feedback-seeking behavior on firm sustainability through product eco-innovation was stronger at a higher level of TMT boundary spanning behavior (*P* = 0.09, *p* < 0.05). However, the direct effect of the interaction on product eco-innovation was not significant, which suggested that the interaction of TMT boundary spanning behavior and feedback-seeking was fully mediated through product eco-innovation on firm sustainability that supported the H_5_. Nevertheless, we did not find full empirical support for the sixth hypothesis as the interaction of TMT boundary spanning behavior and feedback-seeking behavior was significant with both processes eco-innovation and firm sustainability. Additionally, the conditional indirect effects of CEO feedback-seeking behavior on firm sustainability through process eco-innovation was not significant with low TMT boundary spanning behavior (*P* = 0.12, *p* > 0.05).

**TABLE 4:** Bootstrapping-based moderated path analysis.

	First-stage moderation	Second-stage moderation	Direct effect	Indirect effect	Total effect
	P_MX_	P_YM_	P_YX_	P_YM_P_MX_	P_YX_ + P_YM_P_MX_
**CFS → PCEI → Firm sustainability**					
Low TMT boundary spanning	0.02	0.11	0.12	0.01	0.13
High TMT boundary spanning	0.04*	0.23**	0.14*	0.09	0.23**
**CFS → PDEI → Firm sustainability**					
Low TMT boundary spanning	0.02	0.25**	0.16	0.12	0.28
High TMT boundary spanning	0.13	0.13*	0.09*	0.03	0.12*
**Moderating effect of TBS**					
CVA → PCEI → Firm sustainability	0.17*	0.29**	0.19*	0.13**	0.32*
CVA → PDEI → Firm sustainability	0.07	0.17*	0.08	0.09	0.17
CFS → PCEI → Firm sustainability	0.15	0.24**	0.18*	0.12	0.30*
CFS → PDEI → Firm sustainability	0.10*	0.19*	0.21	0.16*	0.37*

^1^Researchers argued that Cronbach’s alpha is pointless and inadequate while testing the reliability of two-item scale. As an alternative, Pearson correlation is more appropriate measure in the regard (Eisinga et al., 2013). *p < 0.05; **p < 0.01.

Results also show that TMT boundary-spanning behavior moderates the mediating link of CEO vision articulation on firm sustainability through product eco-innovation (*P* = 0.13, *p* < 0.01), while the mediating link of CEO feedback-seeking behavior on firm sustainability through process eco-innovation can be moderated with TMT boundary-spanning behavior (*P* = 0.16, *p* < 0.05). Owing to the insignificant estimates for the sixth and seventh hypotheses, the null of these hypotheses cannot be rejected.

## Discussion

Owing to exorbitant economic activities, Asian economies are on the verge of an atmospheric catastrophe. Thus, business sectors should transcend from greenwashing campaigns and veridically transform their core cultural values to environmental healing. To achieve higher sustainable performance, organizations need to transform their eco-deficit culture to eco-surplus culture (Vuong, 2021). Thus, rigorous eco-innovation is required to simultaneously create monetary and environmental value. This study aims to investigate how CEO’s vision articulation and feedback-seeking behavior utilize eco-innovation as a channel to enhance a firm’s sustainability in the presence of top management teams support. Accordingly, we proposed a moderated mediation model in which TMT boundary-spanning behavior moderated the indirect effect of CEO vision articulation and feedback-seeking behavior on the firm’s sustainability through product and process eco-innovation.

First, we find that eco-innovation is an important mechanism that builds the link between CEO vision articulation and firms’ sustainability. Consistent with the upper echelons perspective, it is argued that visionary leaders dynamically thrive for achieving competitive advantage and sustainability by stimulating eco-friendly technological innovation (Arena et al., 2018). Although empirical support for this argument is weak, however, visionary CEOs may enhance environmental innovation and thereby sustainable competitiveness of a firm through employee creativity (Zhou et al., 2018; Mascareño et al., 2020) and radical innovation (Sattayaraksa and Boon-itt, 2018).

On the other hand, CEO feedback-seeking behavior can improve firm sustainability through product eco-innovation only. Humble leaders who consistently seek feedback on their performance may consider environmental concerns before executing their profit-oriented strategies (Jones Christensen et al., 2014). Humility is also associated with responsible leaders who motivate their employees to engage in organizational citizenship behavior for the environment (Han et al., 2019). Additionally, employees feel motivated to speak up under the supervision of humble leaders (Lin et al., 2019), results in a supportive organizational climate (Yuan et al., 2018), and employee innovative behavior (Zhou and Wu, 2018). Thus, the feedback-seeking behavior of CEOs may motivate their employees to indulge themselves in eco-friendly innovation to improve the long-term sustainability of their firms. Self-expansion theory is also applicable in this context as humble CEOs may trigger the self-expansion process of their team members to enhance self-efficacy, creative thinking, and overall task performance (Mao et al., 2016). Nonetheless, humility or feedback-seeking is more relevant for product eco-innovation rather than process eco-innovation. Possibly, the decisions of Chinese CEOs who seek feedback from their colleagues/TMTs are more driven by the market demand for green products rather than cost-saving or technology push (Triguero et al., 2013).

Results are also partially consistent with a paradox perspective that vision articulation and feedback-seeking behavior can coexist in a CEO (Ashford et al., 2018). When CEOs lack a strong vision to influence followers and gain support, then their feedback-seeking behavior may facilitate their aim of engaging employees in green product innovation. However, environmental process innovation focuses on pollution emissions and reduction of non-renewable energy consumption, needs more involvement, and required returns cannot be obtained with a stronger vision. Lastly, we explore the role of TMT boundary-spanning behavior in facilitating visionary CEOs for product eco-innovation and feedback-seeking behavior for process eco-innovation. Based on social capital theory, it is purported that the boundary-spanning behavior of top management teams is a cost-effective way of gaining external resources (Yan et al., 2020) and stimulating innovative performance (Jiao et al., 2019). Furthermore, they help CEOs to adjust their vision according to market sensitivities and provide constructive feedback to humble CEOs through their effective lateral communication networks with external stakeholders to enhance the firm’s long-term sustainability through eco-innovation.

## Implications, Limitations, and Future Research Directions

Our findings make several theoretical and practical implications. We enrich the literature on the linkage between top management behavior and the firm’s green practices for sustainable long-term development. We suggest that environmental innovation is an adequate mechanism to link the goals of visionary and humble CEOs with the firm’s sustainable growth. Although the role of a CEO’s vision to fortify organizational innovation is important, humility may overcome the fragile sense of vision to foster eco-innovation and sustainability. Nonetheless, excessive humility may threaten the leader’s prestige, due to which visionary leadership should be combined for enhancing innovative culture, TMT integration, and organizational sustainability.

Unlike prior studies, we integrate the upper echelons perspective with the social capital perspective by incorporating TMT boundary-spanning behavior in the framework. Organizations should focus on improving the boundary-spanning functions of their top management teams to gain flexible and low-cost external resources to improve firm sustainability through green product and process innovation. Additionally, TMT boundary-spanning behavior extracts resources for visionary CEOs for product eco-innovation and feedback-seeking CEOs for process eco-innovation. Thus, boundary-spanning behavior is a powerful tool to manage both process and product innovation for sustainable organizational development.

Concerning the practical implications of our findings, it is suggested that organizations should focus on the behaviors of CEOs to engage the enterprises in the green innovation of products and processes. The selection of CEOs should be based on their ability to articulate a clear vision and be humble with their followers as these behaviors complement each other to foster sustainable competitiveness. Additionally, corporations should establish effective procedural mechanisms to recognize the integration of TMT boundary-spanning behavior by encouraging them to create valuable resource networks and communicate actively through trial-and-error learning systems. Unfortunately, Asian countries are hard hit by climate change but still, their understanding of corporate sustainability issues is inadequate (Vuong et al., 2021). These firms rarely have a genuine concern for improving social and environmental performance. Their major motivation to pursue CSR activities is either reputational concern or short-term financial interests. It is high time for regulatory bodies to build a more inclusive database on business engagement in sustainability initiatives for increasing public scrutiny.

This study is subject to certain limitations that can provide additional opportunities for future studies. First, this study has utilized multisource cross-sectional data, which did not account for the variation in organizational behavior, processes, and the possibility of reverse causality over time. Thus, the longitudinal time-lagged study may provide more robust estimates. Secondly, our sample size is smaller at an organizational level despite surveying a large number of TMT members. Future studies may consider a large sample size to mitigate the statistical biases associated with a smaller sample size. Thirdly, studies should include national factors or conduct a cross-country study as the cultural background of China may vary from other Asian or Western countries.

## Data Availability Statement

The raw data supporting the conclusions of this article will be made available by the authors, without undue reservation.

## Ethics Statement

The studies involving human participants were reviewed and approved by the Ethics Committee at Jiangsu University. The patients/participants provided their written informed consent to participate in this study.

## Author Contributions

All authors listed have made a substantial, direct, and intellectual contribution to the work, and approved it for publication.

## Conflict of Interest

The authors declare that the research was conducted in the absence of any commercial or financial relationships that could be construed as a potential conflict of interest.

## Publisher’s Note

All claims expressed in this article are solely those of the authors and do not necessarily represent those of their affiliated organizations, or those of the publisher, the editors and the reviewers. Any product that may be evaluated in this article, or claim that may be made by its manufacturer, is not guaranteed or endorsed by the publisher.
